# Long-term results of super-selective trans-catheter embolization of the vesical arteries for the treatment of intractable bladder haematuria

**DOI:** 10.1186/s42155-020-00188-1

**Published:** 2020-12-11

**Authors:** Maria Tsitskari, Stavros Spiliopoulos, Chrysostomos Konstantos, Konstantinos Palialexis, Lazaros Reppas, Elias Brountzos

**Affiliations:** 1 Department of Vascular and Intervenional Radiology, Apollonion Hospital, Lefkotheou street 20, Strovolos, Nicosia, Cyprus; 2 2nd Radiology Department, Division of Interventional Radiology, Attikon University General Hospital, Athens, Greece

**Keywords:** Intractable bladder hematuria, Embolization, Particles, Glue, Gelfoam

## Abstract

**Purpose:**

To evaluate the feasibility, safety and long-term efficacy of super-selective trans-catheter arterial embolization for the management of intractable bladder bleeding.

**Materials and methods:**

The records of 20 patients with intractable haematuria referred urgently for selective arterial embolization after failed conventional therapy, between 2013 and 2018, were retrospectively analyzed. Primary outcomes were technical (cessation of extravasation and/or stasis of flow within the target vessel) and clinical (bleeding control) success. Secondary outcomes included complication and re-intervention rates.

**Results:**

Technical success was 90% (18/20 cases), as in 2 cases, embolization was not feasible. Super-selective embolization of the vesical arteries was feasible in 15/18 cases (83.3%). Selective proximal occlusion of the anterior division of the internal iliac artery was performed in two cases (11%) and embolization of the anterior division after coil blockage of the posterior division was performed in one case (5%). Bilateral and unilateral embolization was performed in 10 and 8 cases, respectively. Peri-procedural mortality rate was 5% (1/18 patients). One possible procedure-related death occurred due to myocardial infarction ten days following non-target embolization of the buttocks and the anterior abdominal wall. Mean time follow up was 35 ± 15 months. Bleeding reoccurred in three patients (16.6%), all successfully managed (one conservatively and two with further embolization). Clinical success was 85% (17/20 cases). During follow up 11 more patients died, due to underlying conditions not related to bleeding or the procedure.

**Conclusions:**

Super-selective angiographic embolization is feasible, safe and effective to control refractory, life threatening bladder bleeding and should be considered as a first line treatment, as to obviate the need for emergency surgery.

## Background

Intractable haematuria is a severe, life-threatening condition. It may have a host of causes such as an underlying malignancy mainly prostate and bladder cancer, inflammatory causes or radiation and drug induced cystitis (Ghahestani and Shakhssalim 2009). Most cases of bladder and prostate haematuria are managed conservatively by irrigation with formalin, silver nitrate or alum solution, intra-vesical hydrostatic pressure, hyperbaric oxygen or endoscopic diathermy. Bladder resection with urinary diversion has traditionally been applied in cases that conservative treatment failed. (Choong et al. 2000) The majority of patients with intractable haematuria are of high surgical risk due to advanced age, various comorbidities and poor clinical status. Over the past decade percutaneous, trans-catheter, arterial embolization has been proposed as an attractive, minimal invasive, alternative treatment for patients suffering from bladder haematuria not responding to conventional, non-invasive treatment. Nonetheless, data regarding the safety and efficacy of the method remain scarce. (Loffroy et al. 2014) We sought to assess the feasibility, safety and long-term efficacy of super-selective, trans-catheter, embolization of the vesical arteries for the management of intractable bladder haematuria in our institution.

## Materials and methods

### Study design

This was a retrospective study not requiring approval by the Hospital’s Ethics and Scientific Committee. The potential risks and benefits were explained and informed consent was obtained from all individual participants included in this study. Records of all patients referred to the Interventional Radiology department for the management of intractable haematuria between 2013 and 2018, were retrospectively reviewed. In our institution, percutaneous embolization, is usually the preferred method of treatment for patients with haematuria non responsive to conservative measures due to its minimal invasiveness compared to surgical management. In total 20 patients [18 men (90%); mean age 76 ± 4 years] who were treated with percutaneous trans-catheter arterial embolization were included in this study. The decision to perform trans-catheter embolization was based on clinical and laboratory evidence of continued bleeding despite adequate conventional therapy. The majority of these patients were hemodynamically stable and were treated on an urgent basis. One patient developed massive hematuria and hemodynamic instability while being treated conservatively and was referred for embolization in the emergency setting.

### Embolization procedure

All procedures were performed under local anaesthesia, after obtaining right common femoral artery access via a 6-Fr vascular sheath. Initially pelvic angiography was performed using a 5 F pigtail catheter to delineate the pelvic arterial anatomy. Selective catheterization and angiography of the internal iliac arteries was then performed using a Tempo Aqua catheter (Cordis Europa N. V, Roden, The Netherlands) or a Sim 1 hydrophilic catheter (Progreat, Terumo Europe NV, Leuven, Belgium). Subsequently, the anterior division of the iliac artery was selectively catheterized and digital subtraction angiography (DSA) was performed (bilaterally) to adequately visualize vesical branches and depict any pathologic vascularity of the urinary bladder and/or a site of active extravasation. Based on angiographic findings super-selective catheterization of the vesical artery was done using a 2.7Fr coaxial micro-catheter (Progreat®, Terumo Europe NV, Leuven, Belgium). Flow directed embolization was performed using microspheres or glue or both. (Table 1) When the main distal branches of the anterior division of the internal iliac artery could not be catheterized super-selectively, the catheter tip was left in the anterior division and embolization was performed from this point using gelfoam or microspheres. The aim was bilateral, super-selective, embolization of the vesical arteries until stasis was achieved even if a clear bleeding site was not detected. Unilateral embolization was preferred only in cases in which selective DSA detected active extravasation from a specific terminal arteriole or the presence of a rather focalized than diffuse vascular pathology, or in cases where bilateral catheterization was not possible.

**Table 1:** Patients demographics and procedural details

No Age (yrs)/Sex	DSA findings	Medical history/clinical status	Artery embolized	Embolic Agent	Technical Success	Complications	Re-interventions	Clinical success
1 78 yrs./M	Rt Active extravasation	Ca bladder, HTC drop, HS	Rt Superior vesical	Glue	Yes	No	No	Yes
2 82 yrs./M	Pathologic vascularity	Ca bladder, HTC drop, HS	Bilateral vesical	Particles 300–500 μm	Yes	No	No	Yes
3 58/M	Hypervascular mass	Metastatic osteosarcoma, HS	Rt anterior division of IIA	Gelfoam	Yes	No	No	Yes
4 62/F	Pathologic vascularity	Irradiation cystitis, HS	Bilateral vesical	Particles 300–500 μm 100–300 μm	Yes	No	No	Yes
5 68/M	Pathologic vascularity	CA bladder, HTC drop, UNS	Bilateral vesical	Glue +distal coil Rt IIA	Yes	No	Same day Bilateral super-selective PVA 300-500 μm	Yes
6 73/F	Pathologic vascularity	Ca bladder, HTC drop, HS	Bilateral vesical	Glue	Yes	YES	No	Yes
7 61/M	Pathologic vascularity	Ca bladder, HTC drop, HS	Lt vesical	Particles 250 μm	Yes	No	No	Yes
8 80/F	Pathologic vascularity	Ca bladder, HTC drop, HS	Bilateral vesical	Particles 500–700 μm	Yes	No	No	Yes
9 67/M	Pathologic vascularity	Ca bladder, HTC drop, HS	Bilateral vesical	Glue	Yes	No	No	Yes
10 69/F	Pathologic vascularity	Irradiation cystitis, HTC drop, HS	Rt vesical	Glue + Particles 300–500 μm	Yes	No	Recurrence at 35 days. Rt vesical microspheres 300–500 μm	Yes
11 70/M	Pathologic vascularity	Ca bladder, HTC drop, HS	Rt anterior division of IIA	Gelfoam	Yes	No	Recurrence at 43 days. Conservative treatment	Yes
12 74/F	Hypervascular mass	Ca bladder, HTC drop, HS	Rt vesical	Particles 300–500 μm	Yes	No	No	Yes
13 65/M	Hypervascular mass	Ca bladder, HTC drop, HS	Bilateral vesical	Particles 500 μm	Yes	No	No	Yes
14 78/F	Pathologic vascularity	Ca bladder, HTC drop, HS	Bilateral vesical	Particles 600 μm	Yes	No	No	Yes
15 75/M	Pathologic vascularity	Ca bladder, Previous surgery, HTC drop, HS	No IIA catheterization	N/A	No	No	N/A	No
16 70/F	Pathologic vascularity	Ca bladder, HTC drop, HS	Bilateral vesical	Glue	Yes	No	No	Yes
17 67/M	Lt active extravasation	Ca bladder, HTC drop, UNS	IIA Posterior division	Coil + particles 40 μm	Yes	Ischemia, MI, death	No	No
18 81/M	Pathologic vascularity	Ca bladder, HTC drop, UNS	Bilateral vesical	Particles 300–500 μm	Yes	PES	No	Yes
19 89/M	Pathologic vascularity	Ca bladder, HTC drop, HS	Lt vesical	Glue	Yes	No	No	Yes
20 82/F	Pathologic vascularity	Ca bladder, HTC drop, HS	No IIA catheterization	N/A	No	N/A	N/A	No

*Ca* cancer, *HTC* haematocrit, *HS* haemodynamically stable, *UNS* haemodynamically unstable, *IIA* internal iliac artery, *MI* myocardial infarction, *PES* post-embolization syndrome

### Definitions, outcomes and follow up

All study outcome measures were defined according to the SIR reporting standards. (Angle et al. 2010) Primary outcome measures were: a. technical success defined as cessation of extravasation and/or stasis of flow within the target vessel on post-embolization DSA and b. clinical success defined as the resolution of signs or symptoms that prompted the embolization throughout the follow up period, without the occurrence of major complications. Secondary endpoints included super-selective target vesical artery catheterization and embolization rates, procedure-related major and minor complication rates and re-intervention rates defined as the rate of additional embolization or open surgical procedures due to recurrence of haematuria. Follow up was assessed by reviewing the medical records of all patients and by telephone interview in case of missing data.

## Results

**Fig. 1:**
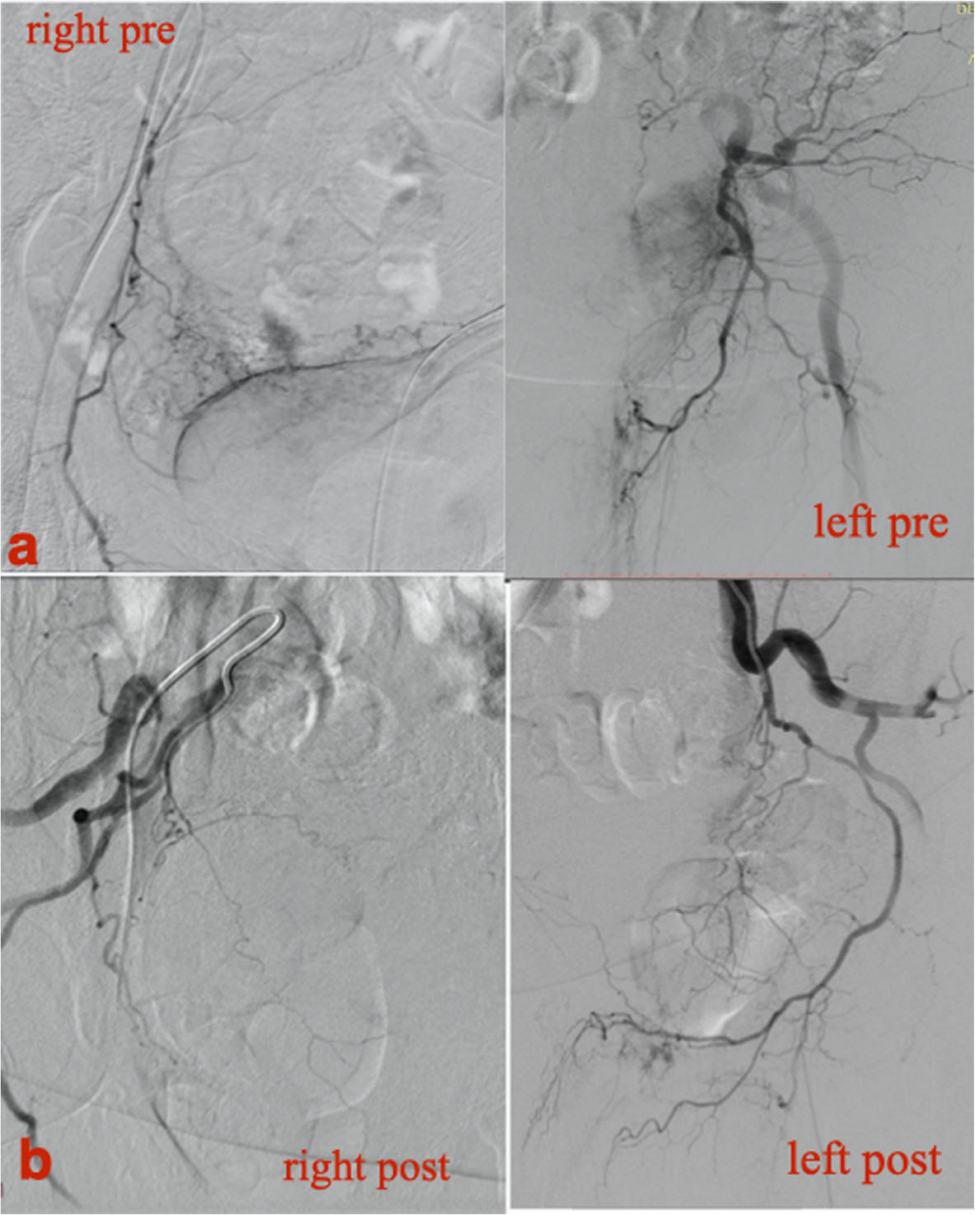
Haemodynamically unstable 72-year old male patient with inoperable urinary bladder carcinoma and multiple episodes of haematuria not responding to conventional therapy. a. Selective DSA from the anterior division of the IIA bilaterally, demonstrating pathologic vascularity of the bladder. b Final DSA following micro-particle embolization (500-700 μm) demonstrating almost complete devascularization of the bladder. Patient remained asymptomatic without clinical relapse

**Fig. 2:**
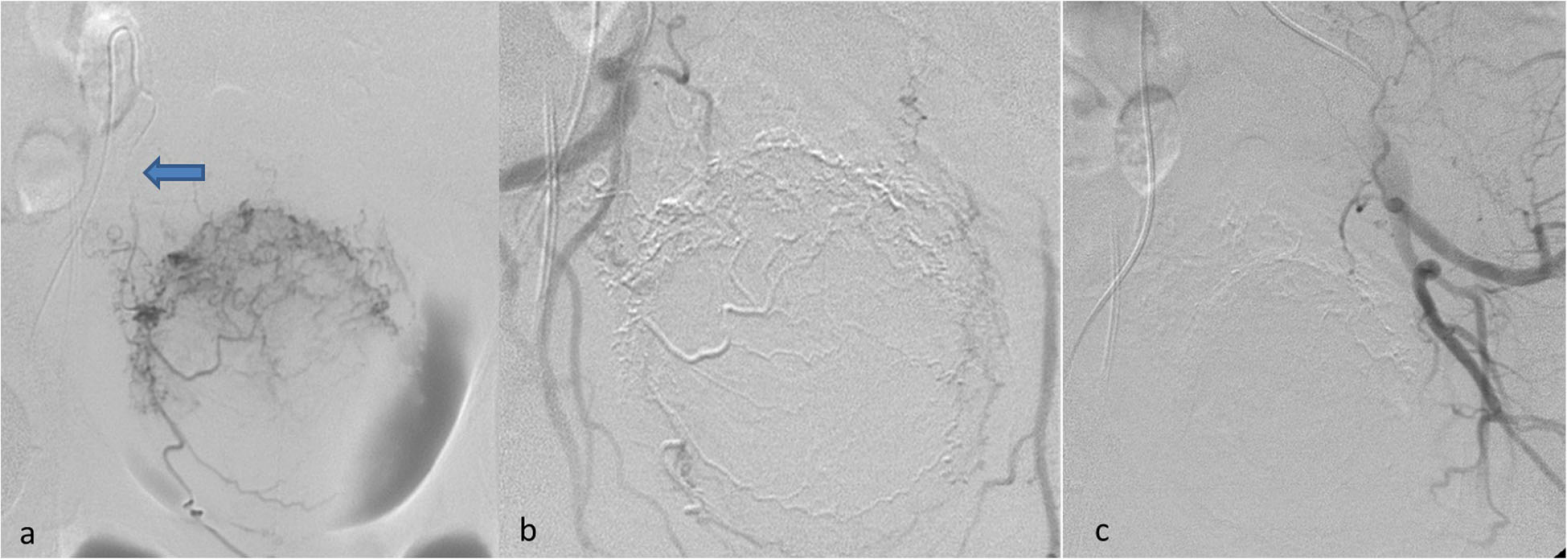
Haemodynamically stable 84-year-old male patient suffering from with inoperable urinary bladder carcinoma and multiple episodes of haematuria not responding to conventional therapy. **a** Super-selective DSA with a micro-catheter within the right superior vesical artery (blue arrow) demonstrating pathologic vascularization of the bladder. **b** and **c** DSA following bilateral superior vesical artery glue embolization demonstrating complete devascularisation of the bladder. No bleeding relapse occurred

During the study period a total of 22 angiography procedures were performed (including re-interventions) for embolization of severe intractable bladder hematuria in 20 patients. Most patients were at high operative risk due to advanced age and comorbidities as the underlying pathology was urinary bladder cancer (*n* = 16 patients), prostate cancer (n = 1), metastatic osteosarcoma of the urinary bladder (n = 1) and post irradiation cystitis (*n* = 2). (Table 1) Angiography findings included diffuse increased pelvic vascularity/neovascularity in 15 cases, active extravasation in two patients and localized hypervascular mass-like lesion in three patients. In one of the three patients with mass-like lesions the presence of an AVF was also depicted. Technical success was 90% (18/20 cases), as in 2/20 cases (10%), embolization was not technically feasible. In one patient catheterization of the internal iliac arteries was not achieved after multiple attempts due to severe atherosclerosis and tortuosity of the iliac arteries, while in the second case catheterization was not feasible due to altered vascular anatomy following previous prostatectomy and partial bladder resection, not enabling safe embolization. Both patients were managed conservatively and died after 1 and 2 months respectively. Super-selective distal embolization of the vesical arteries was performed in 15 out of 18 cases (83.3%) using PVA foam particles (Fig. 1) ranging from 100 to 500 μm (Cook Medical, UK) or PVA hydrogel particles ranging from 300 to 700 μm (Bead Block; Terumo Europe NV, Leuven, Belgium) or glue (GLUEBRAN®2,GEM S.r.l, LU,Italy) (Fig. 2), or a combination of particles and glue. In 3/18 patients (16.6%) who underwent trans-catheter embolization, super-selective catheterization of the vesical arteries was not feasible. Selective proximal Gelfoam sponge embolization of the anterior division of the internal iliac artery was performed in 2 out of these 3 patients while in one patient selective particle embolization was performed after coil protection of the posterior division. Embolization was performed bilateral in 10/18 patients (55.5%) and unilateral in 8/18 (44.43%) based to angiographic findings and technical feasibility. In 3 patients bilateral embolization was desirable but not possible.

**Fig. 3:**
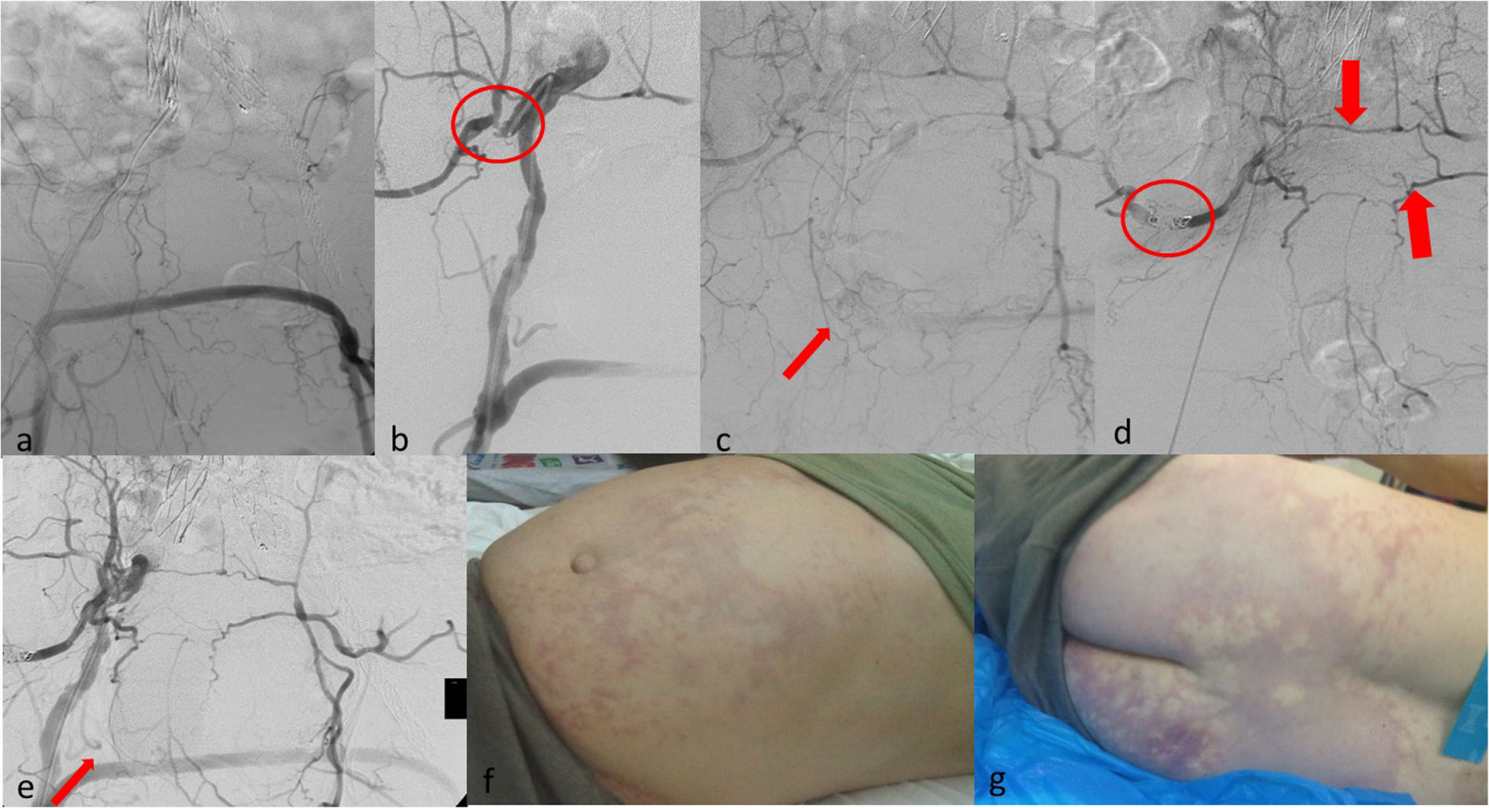
Haemodynamically unstable 61-year old male patient with history of inoperable bladder cancer, radiotherapy previous EVAR, fem-fem bypass graft and intractable haematuria not responding to any conservative method. **a** DSA depicting previous unilateral EVAR and extra-anatomic fem-fem bypass. **b** Selective catheterization of the right internal iliac artery (IIA) demonstrating a significant stenosis of its origin (circle). **c** DSA with the catheter at the origin of the IIA demonstrating a blush of contrast at the site of bleeding (arrow). **d** As the anterior division of the IIA could not be selectively catheterized, protective coil embolization of the posterior division (circle) and life-saving particle embolization (40 μm for better penetration) from the origin of the IIA was performed. Note the anastomosis between the right and the left middle and lateral sacral arteries (arrows). **e** After the delivery of 1 vial of micro-particles no blush was evident (arrow). **f** and **g** 30 mins after embolization the patient developed severe pain and marked ischemia of the buttock and the anterior abdominal wall. He was managed by the anaesthesiology team and died of myocardial infarction 10 days after index procedure

Mean follow up period was 35 ± 15 months. Clinical success rate was 85% (17/20 cases) as there was one procedure-related death (1/18 patients; 5%) following non- target embolization of the buttocks and the anterior abdominal wall after proximal embolization of the anterior division of the internal iliac artery using embolic microspheres of 40 μm (Embospheres, Merit Medical Systems, Inc.) and coil embolization (Nester, Cook Medical) for the preservation of the posterior IIA division (Fig. 3). This patient developed ischemic changes at the anterior abdominal wall and buttock area (Fig. 3) and died due to myocardial infarction 10 days later. Bleeding recurrence was noted in 3/18 cases (16.6%). In one patient re-bleeding occurred 5 h after initial embolization and was controlled by repeated super-selective embolization. In the remaining two patients re-bleeding occurred 35 and 43 days after initial embolization. One was successfully managed conservatively and the other with successful repeat superselective embolization. During the follow up period 11 more patients died. Two of these patients died due to heart disease, one due to pulmonary embolism, one due to stroke and seven due to the underlying malignancy.

Minor complications were noted in 2/18 (11%) of the cases as two patients experienced post embolization syndrome, which was managed conservatively only with analgesics and antipyretics and resolved within 10 days.

Outcomes of our study are summarized in Table 2.

**Table 2:** Study’s outcomes

**Technical success (%)**	18/20 (90)
**Clinical success (%)**	17/20 (85)
**Re-embolization (%)**	2/18 (100)
**Re-bleeding**	
Early (less than 1 month) (%)	1/18 (5.5)
Late (more than 1 month) (%)	2/18 (11.1)
**Complications**	
Minor (%)	2/18 (11.1)
Major (%)	1/18 (5.5)
**Mortality**	
Peri-procedural (%)	1/18 (5.5)
Overall (during follow up)	12/18 (66.6)
**Cause of death**	
Procedure-related	1/18 (5.5)
Underlying malignancy	7/18 (38.8)
Other pathology	4/18 (22.2)

## Discussion

Refractory bladder haemorrhage represents a major clinical problem treated on an emergency bases. According to the literature, various conservative measures available result in a response rate ranging from 50% to 100% (Ghahestani and Shakhssalim 2009; Choong et al. 2000). Surgical treatment is associated with high morbidity and mortality since most of these patients suffer from serious comorbidities. Percutaneous, trans-catheter, arterial, embolization (TAE) is an alternative, minimally invasive therapeutic option with good results and a lower complication rate compared to conventional surgical approach. (Loffroy et al. 2014)

Review of the literature reveals only few small case series or sporadic case reports reporting results from endovascular embolization for severe haematuria. [Table 3] Most of these studies support the safety and efficacy of the technique in short and mid-term follow up and present a low complication rate. (Loffroy et al. 2014) The herein presented outcomes indicate that super-selective, vesical artery TAE is feasible in the majority of the cases and achieves very satisfactory long-term clinical success rates of 85%.

**Table 3:** Published series of embolization for intractable hematuria

Study / year	Patient No	Follow up (months)	Superselective embolization (%)	Bilateral embolization (%)	Technical Success (%)	Complication rate(%)	Clinical success(%)
(Rastinehad et al. 2008)	10	20	100	100	100	10	100
(Prasad et al. 2009)	11	2	90,9%	90,9	100	9,1	90,9
(G. Liguori et al. 2010)	44	10,5	100%	100	100	27	82
(A. Delgal et al. 2010)	20	16	88,9%	72,2	90	10	100
(Korkmaz et al. 2016)	18	18	N/A	N/A	88	27	100

Rebleeding represents the weak point of the method and in our series rebleeding was encountered in 3/18 (16%) of the patients. This is in keeping with the lower reported rates which range between 10 to 28%. (Delgal et al. 2010) Earlier studies describe mainly nonselective unilateral internal artery embolization, associated with high complication and re-bleeding rate (Liguori et al. 2010; Hietala 1978; Pisco et al. 1989). Overall we treated 10 patients (55.5%) with bilateral super-selective embolization of the vesical arteries and only one of these patients (10%) suffered bleeding recurrence, 5 h later following initial TAE. As a result, the patient underwent second bilateral super-selective TAE using microspheres with successful clinical outcome. On the contrary 8 patients underwent unilateral embolization and rebleeding was noticed in 2 patients (25%), at 35 and 43 days respectively, following initial treatment. One managed conservative and one with further unilateral embolization of the same vessel. Bilateral approach seems to decrease the rate of early re-bleeding which is attributed to the rich collateral blood supply to the internal iliac artery. (Liguori et al. 2010; Hietala 1978; Rastinehad et al. 2008; Ozono et al. 1988; Prasad et al. 2009) We do argue that bilateral embolization may lead to more durable response although is hard to support it due to lack of technique standardization and the small number of patients treated.

Super-selective embolization seems to have fewer complications. (Hietala 1978; Pisco et al. 1989) Delgal et al. reports the safety and efficacy of super-selective embolization with particles in 11 patients. (Delgal et al. 2010) We also performed super-selective embolization with particles or glue or a combination of both without complications. The sole major complication in this series was encountered following TAE from the orifice IIA anterior division using microspheres after coil blockage of the posterior division. Selective distal catheterization of the anterior division was not feasible due to severe stenosis in its proximity. Few hours later the patient developed severe pain from gluteal and anterior abdominal wall necrosis. He died ten days later due to myocardial infarction. On a retrospect, this patient had a history of peripheral arterial disease, previous endovascular aortic aneurysm repair and fem-fem bypass, with a rich collateral network due to the underlying severe atherosclerosis that was over looked. Moreover small sized particles of 40 μm were used aiming in better penetration. All the above contributed to this disastrous complication could definitely be prevented if the angiographic findings were cautiously evaluated. The use of 300- to 500 μm particles would have certainly been a better choice, as it has been reported to decrease the complication rate given a lower risk of non-targeted embolization and/or tissue devascularisation. Surgery was not an option for this patient due to severe comorbidities.

The use of different embolization materials has been reported like coils, glue, particles, alcohol and gelfoam without any actual benefit of a specific agent over another. Most of the authors used particles regardless the selectivity of embolization. (Delgal et al. 2010; Liguori et al. 2010) In the majority of the cases we used particles, glue or a combination of both. There are only few reports of embolization with the use of cyanoacrylate glue in the literature. Delgal, et al., reported cyanoacrylate glue embolization of the feeding branch in patients with angiographic evidence of contrast extravasation. In this study, n-butyl-2-cyanocrylate glue mixed with ultra-fluid lipiodol (1:3 ratio) was used, alone or in combination with other embolic agents in 7 patients, regardless of the angiographic findings. There were no complications observed associated with the use of glue.

According to the literature and the author’s opinion, bilateral superselective approach when possible seems to be the desired treatment option when no site of active bleeding is identified. However definite conclusions about the best technique and embolization agent to optimize the technique efficiency cannot be drawn yet. Unilateral embolization can be efficient in selected cases, especially when angiography depicts bleeding from a terminal arteriole or a focalized hypervascularity rather than diffuse disease. (Delgal et al. 2010) An attempt to embolize one of the vesical arteries or even branches of the IIA should be made, as flow reduction could sometimes suffice as to stop the bleeding or incite clinical improvement.

Limitations of the present study include an inherent external validity bias, the small number of patients investigated due to the single-centre design and the lack of technique standardization. Moreover, the limited number of subjects included did not enable sub-group analysis as to identify possible factors influencing outcomes such as clinical presentation, embolic material, bilateral versus unilateral embolization etc. Finally, as this was a retrospective study some cases might have not been identified.

## Conclusion

Conclusively, according to this retrospective study, palliative, super-selective TAE was proven feasible, safe and effective in controlling refractory bladder bleeding. It is a life-saving procedure, less-invasive than surgery, which resulted in very satisfactory technical success, peri-procedural morbidity and mortality rates, as well as sustained long-term control of haematuria with low re-bleeding rates. This procedure should be considered as an alternative treatment option in selected patients not responding to conservative treatment, as to obviate the need for surgery. However, as data in then literature remain limited, further prospective comparative studies are required in order to validate these results.

## Data Availability

The datasets generated during and/or analysed during the current study are available from the corresponding author on reasonable request.

## References

[CR1] Angle JF, Siddiqi NH, Wallace MJ (2010). Quality improvement guidelines for percutaneous Transcatheter embolization Society of Interventional Radiology Standards of practice committee. J Vasc Interv Radiol.

[CR2] Choong SKS, Walkden M, Kirby R (2000). The management of intractable haematuria. BJU Int.

[CR3] Delgal A, Cercueil JP, Koutlidis N (2010). Outcome of Transcatheter Arterial Embolization for Bladder and Prostate Hemorrhage J Urol.

[CR4] Ghahestani SM, Shakhssalim N (2009). Palliative Treatment of Intractable Hematuria in Context of Advanced Bladder Cancer. Urol J.

[CR5] Hietala SO (1978). Urinary bladder necrosis following selective embolization of the internal iliac artery. Acta Rad Diagn.

[CR6] Korkmaz M, Sanal B, Aras B (2016). The short- and long-term effectiveness of transcatheter arterial embolization in patients with intractable hematuria. Diagn Interv Imaging.

[CR7] Liguori G, Amodeo A, Mucelli FP, Patel H, Marco D, Belgrano E, Trombetta C (2010). Intractable haematuria: long-term results after selective embolization of the internal iliac arteries. BJU Int.

[CR8] Loffroy R, Pottecher P, Cherblanc V (2014). Current role of transcatheter arterial embolization for bladder and prostate hemorrhage. Diagn Interv Imaging.

[CR9] Ozono S, Hirao Y, Babaya K (1988). Transcatheter arterial embolization of vesical artery in the treatment of invasive bladder cancer. Eur Urol.

[CR10] Pisco JM, Martins JM, Correia MG (1989). Internal iliac artery: embolization to control hemorrhage from pelvic neoplasms. Radiology.

[CR11] Prasad V, Sacks BA, Kraus S (2009). Embolotherapy for lower urinary tract hemorrhage. J Vasc Interv Radiol.

[CR12] Rastinehad AR, Caplin DM, Ost MC (2008). Selective arterial prostatic embolization (SAPE) for refractory hematuria of prostatic origin. Urology.

